# Simultaneous Differentiation of the N1 to N9 Neuraminidase Subtypes of Avian Influenza Virus by a GeXP Analyzer-Based Multiplex Reverse Transcription PCR Assay

**DOI:** 10.3389/fmicb.2019.01271

**Published:** 2019-06-07

**Authors:** Sisi Luo, Zhixun Xie, Jiaoling Huang, Zhiqin Xie, Liji Xie, Minxiu Zhang, Meng Li, Sheng Wang, Dan Li, Tingting Zeng, Yanfang Zhang, Qing Fan, Xianwen Deng

**Affiliations:** ^1^Guangxi Key Laboratory of Veterinary Biotechnology, Guangxi Veterinary Research Institute, Nanning, China

**Keywords:** avian influenza virus, neuraminidase, GeXP analyzer, multiplex PCR, differentiation diagnosis

## Abstract

To date, nine neuraminidase (NA) subtypes of avian influenza virus (AIV) have been identified in poultry and wild birds. Rapid and effective methods for differentiating these nine NA subtypes are needed. We developed and validated a rapid, sensitive, and robust method utilizing a GeXP analyzer-based multiplex RT-PCR assay and capillary electrophoresis for the simultaneous differentiation of the N1 to N9 subtypes in a single-tube reaction. Ten pairs of primers–nine subtype-specific pairs and one pan-AIV pair–were screened and used to establish the GeXP multiplex RT-PCR assay. A single subtype was detected using the developed GeXP assay; the N1 to N9 AIV subtypes individually generated two target peaks: the NA subtype-specific peak and the general AIV peak. Different concentrations of multiplexed subtypes were tested with this GeXP assay and the peaks of the corresponding NA subtypes were generated, suggesting that this GeXP assay is useful for identifying NA subtypes in mixed samples. Moreover, no peaks were generated for other important avian viruses, indicating negative results and validating the lack of cross-reactions between AIV subtypes and other avian pathogens. RNA templates synthesized through *in vitro* transcription were used to analyze the sensitivity of the assay; the limit of detection was 100 copies per reaction mixture. The results obtained from clinical samples using this GeXP method were consistent with the results of the neuraminidase inhibition (NI) test, and the ability of the GeXP assay to identify mixed infections was superior to amplicon sequencing of isolated viruses. In conclusion, this GeXP assay is proposed as a specific, sensitive, rapid, high-throughput, and versatile diagnostic tool for nine NA subtypes of AIV.

## Introduction

Four types of influenza viruses, designated influenza A virus, influenza B virus, influenza C virus, influenza D virus, have been identified (Yu et al., 2017). Influenza A virus infects birds, humans, swine, and many other mammalian species. Avian influenza virus (AIV) is a negative-sense, segmented, single-stranded, enveloped RNA virus belonging to influenza A virus in the Orthomyxoviridae family. Two major antigens of AIV, hemagglutinin (HA) and neuraminidase (NA), allow subtype classification based on antigenic diversity. Sixteen HA and nine NA subtypes have traditionally been identified in poultry and wild birds, and the 17th and 18th HA subtypes and 10th and 11th NA subtypes were recently discovered in bats (Tong et al., 2012, 2013). Four influenza-driven pandemics occurred in the 20th century (H2N2 in 1957, H3N2 in 1968, and H1N1 in 1918 and 2009), in which millions of people perished. China is recognized as a geographical area with an environment suitable for the emergence of novel influenza viruses (Su et al., 2015). New strains of AIV, including at least six subtypes of AIV (H5N1, H5N6, H7N9, H10N8, H9N2, and H6N1), are currently circulating widely in China or have resulted in occasional human infections in recent decades. The first confirmed human case of infection with the highly pathogenic H5N1 strain of AIV was reported in Hong Kong in 1997, the first recognized case of virus transmission directly from poultry to humans; a second outbreak of H5N1 viruses occurred in 2003, and continuing occurrences have been reported (Yamaji et al., 2015). H5N1 viruses have currently spread to avian species in many countries and threaten animal and human health (Li et al., 2004). In May 2014, China formally confirmed the first human infection with a novel H5N6 strain of AIV in Sichuan Province, and H5N6 has replaced H5N1 as the dominant epidemic strain in China. Currently, H5N6 strains of AIV are the most prevalent, widespread and harmful subtypes observed in Asia and Southeast Asia [World Organization for Animal Health (OIE)] (Sun et al., 2018). Up to March 31, 2013, the H7N9 subtype of AIV was not known to infect humans. Since the first outbreak of human infection with the H7N9 subtype of AIV was identified, five waves of human infection have occurred in China (Shen and Lu, 2017). Originally, H7N9 viruses were non-pathogenic in chickens, but mutated to a highly pathogenic strain in early 2017 and caused severe disease outbreaks in chickens. A bivalent inactivated H5/H7 vaccine for chicken was introduced in September 2017, and the rate of H7N9 virus isolation in poultry decreased substantially after vaccination. More importantly, only three H7N9 cases were reported in humans between October 1, 2017 and September 30, 2018, indicating that the vaccination of poultry successfully eliminated human infection with the H7N9 virus (Zeng et al., 2018). In December 2013, China formally confirmed the first human infection with an avian influenza A (H10N8) virus in Jiangxi Province (To et al., 2014). The H9N2 virus acts as a gene donor for H7N9 and H10N8 viruses, and the role of the H9N2 virus in enabling human infection by wild bird influenza viruses deserves further study (Chen et al., 2014). A case of human infection with an H6N1 virus was reported for the first time in Taiwan in June 2013 (Wang et al., 2015). Based on current evidence, H10N8 and H6N1 AIV infection patterns indicate that this case was most likely sporadic (Qi et al., 2014). However, AIV has pandemic potential and is a global concern. The development of appropriate and effective methods for the accurate diagnosis and tracking of AIV is required to control economic losses due to AIV.

Currently, surveillance to prepare for influenza A virus pandemics is conducted worldwide. Oral and cloacal swabs are collected and inoculated into the allantoic cavity of specific pathogen-free (SPF) chickens for the propagation of AIV. Allantoic fluid is then harvested and examined for HA activity with an HA assay. HA-positive samples are examined further using a hemagglutination inhibition (HI) assay to determine HA subtypes. The HI assay is the most commonly used method for the initial identification of HA subtypes in epidemiological surveys of AIVs. Many tests have been reported to identify HA subtypes, but few are able to identify NA subtypes. After the HA subtype is identified, the NA subtype is usually determined by amplicon sequencing, but an effective, convenient and simple method for identifying the nine NA subtypes is unavailable. The traditional method for NA subtyping is a neuraminidase inhibition (NI) assay with cultured AIV; indeed, this method is the gold standard suggested by the OIE. However, NI assay are more complex and time-consuming than the HI test and require an array of high-quality monospecific antisera and reference antigens. Thus, this method is used less frequently in many labs. Rapid and convenient molecular diagnostic assays for the detection of the nine NA subtypes have been developed. Currently, several reports have described the subtyping of the nine NAs using conventional RT-PCR, all of which used nine pairs of NA-specific primers to amplify NA genes in separate reactions, with nine reactions and nine tubes subsequently subjected to agarose gel electrophoresis (Fereidouni et al., 2009; Qiu et al., 2009; Tsukamoto et al., 2009). Some studies improved and simplified the procedure for subtyping the nine NAs using real-time fluorescent RT-PCR with different fluorophores, which decreased the number of reactions from 9 to 3 or 4 (Huang et al., 2013; Sun et al., 2017). However, few methods enable the simultaneous detection of all nine NA subtypes in a single tube. In this study, we developed a method for simultaneously differentiating the N1 to N9 subtypes in a single reaction, thus decreasing the number of reactions from 9 to 1.

GenomeLab Gene Expression Profiler (GeXP) technology represents a novel platform for high-throughput nucleic acid detection based on multiplex RT-PCR assays and an analysis of amplicons size using capillary electrophoresis, a method that is capable of differentially assessing the expression profile of up to 30 genes in one tube by converting amplification with multiple primers to amplification with a pair of universal primers. The GeXP analyzer has been widely adopted in veterinary diagnostics and medical examinations, and displays high sensitivity and specificity (Hu et al., 2012; Li et al., 2012; Zeng et al., 2015; Zhang et al., 2015; Fan et al., 2017). Currently, a method for the rapid and high-throughput diagnosis of nine NA subtypes is urgently needed. This study aimed to develop a rapid, sensitive, and robust method for differentiating the nine NA subtypes of AIV.

## Materials and Methods

### Primer Design

The sequences of the nine NA genes and the M gene were obtained from the Influenza Sequence Database^1^. Analyses of sequences from representative strains of nine NA subtypes of AIV were conducted and conserved regions were identified using Lasergene 8.0 software. The primers were designed in relatively conserved regions, and primer parameters, such as the annealing temperature, mismatch and dimerization, were evaluated using Primer Premier 5.0 software. The primer sequences were subjected to an *in silico* BLAST analysis in the nucleotide database (NCBI) to evaluate specificity. Chimeric primers consisted of two parts: a designed gene-specific primer and a universal tag primer; the universal forward and reverse tag sequences were attached to the 5′ end of the designed specific primers. Generally, the size of the designed amplicons for GeXP was 105–350 bp without the universal tags and 142–387 bp with the universal tags. Ten pairs of primers with universal tags were finally chosen (Table 1) from the initial evaluation panel of 30 primer pairs, including nine pairs of subtype-specific primers targeting the AIV NA genes and one pair of pan-AIV primers targeting the M gene, a gene conserved across all AIV subtypes. All primers were synthesized and purified by Invitrogen (Guangzhou, China).

**Table 1:** Primer information for the GeXP assay.

Gene#	Gene name	Forward primer (5′-3′)	Reverse primer (5′-3′)	GenBank accession no.	Position	size (bp)
1	N1	AGGTGACACTATAGAATA GGTGTTTGGATCGG**R**AGAAC	GTACGACTCACTATAGGGA TCAACCCAGAA**R**CAAGGTC	KJ907641.1	1006-1025 1214-1196	246
2	N2	AGGTGACACTATAGAATA TTGGGTGTTCCGTTTCA	GTACGACTCACTATAGGGA CCATCCGTCATTACTAC	KF768231.1	506-522 750-734	282
3	N3	AGGTGACACTATAGAATA TTCCCAATAGGAACAGC**Y**CCAGT	GTACGACTCACTATAGGGA TTCTCCATGATTT**R**ATGGAGTC	CY129336.1	487-509 667-646	218
4	N4	AGGTGACACTATAGAATA CAGA**Y**AAGGA**Y**TCAAATGGTGT	GTACGACTCACTATAGGGA CATGGTACAGTGCAATTCCT	CY133359.1	1151-1172 1265-1246	152
5	N5	AGGTGACACTATAGAATA GTGAGGTCATGGAGAAAGCA	GTACGACTCACTATAGGGA TGG**Y**CTATTCATTCC**R**TTCCA	CY196019.1	646-665 906-886	298
6	N6	AGGTGACACTATAGAATA CACTATAGATCC**Y**GA**R**ATGATGACC	GTACGACTCACTATAGGGA GGAGTCTTTGCTAAT**W**GTCCTTCCA	KR919741.1	879-903 1080-1056	239
7	N7	AGGTGACACTATAGAATA GACAG**R**AC**W**GCTTTCAGAGG	GTACGACTCACTATAGGGA GTTGCGTTGTCATTATTTCC	CY167234.1	455-474 612-593	195
8	N8	AGGTGACACTATAGAATA AGGGAATACAATGAAACAGT	GTACGACTCACTATAGGGA TGCAAAACCCTTAGCATCACA	JX304764.1	171-190 288-308	175
9	N9	AGGTGACACTATAGAATA CGCCCTGATAAGCTGGCCACT	GTACGACTCACTATAGGGA ACAGGCCTTCTGTTGTACCA	CY129263.1	472-492 642-623	208
10	AIV-M	AGGTGACACTATAGAATA AGGCTCTCATGGAGTGGCTA	GTACGACTCACTATAGGGA TGGACAAAGCGTCTACGCTG	MH341597.1	119-138 242-223	161

*Universal tag sequences are underlined. Bold type shows degenerate sites. R, A/G; Y, C/T; W, A/T.*

### Preparation of Viruses and cDNAs

The reference isolates of the N1 to N9 subtypes of AIV and several important avian pathogens [newcastle disease virus (NDV), infectious bronchitis virus (IBV), infectious laryngotracheitis virus (ILTV), avian reovirus (ARV), and fowl adenovirus 4 (FAdV4)] were used to assess the specificity of the GeXP assay (Table 2). Viral RNA/DNA was extracted from 200 μL of allantoic fluid of various virus stocks using an EasyPure Viral DNA/RNA kit (TransGen) according to the manual and eluted in 35 μL of nuclease-free water. Reverse transcription was performed in 50 μL reaction mixtures containing 10 μL of 5× M-MLV buffer, 0.6 μL of 40 U/μL RNase inhibitor, 2 μL of 10 mM dNTPs, 1 μL of 200 U/μL M-MLV reverse transcriptase (Takara), 1.5 μL of a 25 μM stock of the 12 bp primer (5′- agcgaaagcagg -3′), and 35 μL of extracted RNA and were then incubated at 42°C for 1 h and stored at −20°C until needed.

**Table 2:** Reference strains used in the GeXP assay.

Number	Strain designation	Source	GeXP assay	Identification of isolate subtype
1	A/Duck/Guangxi/030D/2009 (H1N1)	GVRI	N1	N1
2	Duck/Guangxi/1/04 (H5N1)	GVRI	N1	N1
3	Chicken/Guangxi/1/04 (H5N1)	GVRI	N1	N1
4	A/Duck/Guangxi/GXd-5/2010 (H6N1)	GVRI	N1	N1
5	A/Sparrow/Guangxi/GXs-1/2012 (H1N2)	GVRI	N2	N2
6	A/Duck/Guangxi/GXd-2/2012 (H1N2)	GVRI	N2	N2
7	A/Duck/Guangxi/M20/2009 (H3N2)	GVRI	N2	N2
8	A/Duck/Guangxi/N42/2009 (H3N2)	GVRI	N2	N2
9	A/Chicken/Guangxi/045C2/2009 (H4N2)	GVRI	N2	N2
10	A/Duck/Guangxi/125D17/2012 (H4N2)	GVRI	N2	N2
11	A/Duck/Guangxi/GXd-2/2009 (H6N2)	GVRI	N2	N2
12	A/Chicken/Guangxi/DX/2008 (H9N2)	GVRI	N2	N2
13	A/Chinese Francolin/Guangxi/020B7/2010 (H9N2)	GVRI	N2	N2
14	A/Duck/HK/77/76 (H2N3)	UHK	N3	N3
15	A/Duck/HK/876/80 (H10N3)	UHK	N3	N3
16	A/Shorebird/Delaware/168/06 (H16N3)	UC	N3	N3
17	A/Turkey/Ontario/6118/68 (H8N4)	UHK	N4	N4
18	A/Duck/Guangxi/GXd-1/2009 (H6N5)	GVRI	N5	N5
19	A/Duck/HK/862/80 (H12N5)	UHK	N5	N5
20	A/Gull/Md/704/77 (H13N5)	UHK	N5	N5
21	A/Mallard/Astrakhan/263/82 (H14N5)	UC	N5	N5
22	A/Pigeon/Guangxi/020P/2009 (H3N6)	GVRI	N6	N6
23	A/Duck/Guangxi/175D12/2014 (H3N6)	GVRI	N6	N6
24	A/Duck/Guangxi/070D/2010 (H4N6)	GVRI	N6	N6
25	A/Duck/Guangxi/101D18/2011 (H4N6)	GVRI	N6	N6
26	A/Duck/Guangxi/149D24/2013 (H4N6)	GVRI	N6	N6
27	A/Duck/Guangxi/GXd-4/2009 (H6N6)	GVRI	N6	N6
28	A/Duck/Guangxi/GXd-7/2011 (H6N6)	GVRI	N6	N6
29	A/Chicken Guangxi/129/2013 (H6N6)	GVRI	N6	N6
30	A/Duck/42846/07 (H7N7) RNA	UP	N7	N7
31	A/Goose/Guangxi/020G/2009 (H3N8)	GVRI	N8	N8
32	A/Duck/Guangxi/GXd-6/2010 (H6N8)	GVRI	N8	N8
33	A/Duck/Guangxi/113/2012 (H6N8)	GVRI	N8	N8
34	A/Chicken/QT35/98 (H5N9) RNA	UP	N9	N9
35	A/Duck/PA/2099/12 (H11N9)	UP	N9	N9
36	A/Shearwater/Western Australia/2576/79 (H15N9)	UC	N9	N9
37	B/Guangxi/1418/15	GXCDC	**–**	Influenza B virus
38	B/Guangxi/1470/15	GXCDC	**–**	Influenza B virus
39	NDV F48	CIVDC	**–**	NDV
40	IBV M41	CIVDC	**–**	IBV
41	ILTV Beijing strain	CIVDC	**–**	ILTV
42	ARV S1133	CIVDC	**–**	ARV
43	FAdV4-GX001	GVRI	**–**	FAdV4

*GVRI, Guangxi Veterinary Research Institute, China; UHK, University of Hong Kong, China; UP, University of Pennsylvania, United States; UC, University of Connecticut, United States; CIVDC, China Institute of Veterinary Drug Control; GXCDC, Guangxi Provincial Center for Disease Control and Prevention, China.*

### Evaluation of the Designed Primers Using the GeXP Mono-RT-PCR Assay

The N1 to N9 subtypes of AIV and other important avian viruses (NDV, IBV, ILTV, ARV, and FAdV4) were detected using the GeXP mono-RT-PCR assay to validate the ability of each primer pair to identify the NA subtype. The PCR master mix was prepared in a 20 μL volume containing 4 μL of 5 × GeXP PCR buffer (containing the fluorophore-labeled forward universal primer and the unlabeled reverse universal primer, AB Sciex), 4 μL of 25 mM MgCl_2_, 1 μL of 2.5 U/μL JumpStart Taq DNA polymerase (Sigma), 2 μL each of 200 nM solutions of the forward and reverse primers, 2 μL of transcribed cDNA and 7 μL of nuclease-free water. Since the fluorescent dye is light-sensitive, each PCR should be prepared in dimly lit conditions. PCR was performed with the following cycling conditions: initial denaturation at 95°C for 5 min; followed by 30 cycles at 95°C for 30 s, 50°C for 30 s, and 72°C for 40 s; and a final extension at 72°C for 5 min.

### Separation Using Capillary Electrophoresis and Fragment Analysis

PCR products were separated and detected using a GenomeLab GeXP genetic analysis system (Beckman Coulter) through capillary electrophoresis, according to previously described protocols (Rai et al., 2009). After the amplified fragments were separated, the peaks were initially analyzed using the fragment analysis module of the GeXP system software and matched to the appropriate genes. The peak height for each gene was reported in the electropherogram.

### Evaluation of the Ability of Multiplex Primers to Amplify Single and Multiple Templates Using the GeXP Multiplex RT-PCR Assay

We next selected the optimal primers validated in the GeXP mono-RT-PCR assay for each subtype as candidates for GeXP multiplex RT-PCR. Nine pairs of subtype-specific primers and one pan-AIV primer were combined in a reaction mixture (2 μL each of 200 nM primer stocks), the other reagents and protocols were the same as those used in the GeXP mono-RT-PCR assays. If certain primers did not amplify their target NA subtype after combination, other primers from the candidate primer pools were substituted until the optimal combination of ten primer pairs was identified, ensuring that each primer pair amplified a specific product without cross-reactions. Ten pairs of primers were ultimately selected for the GeXP multiplex RT-PCR assay (Table 1). A single AIV subtype (N1 to N9 of the reference strains, Table 2), random mixtures of multiple AIV subtypes (such as N1+N2, N6+N8+N9, and N3+N4+N5+N7) and other important avian viruses (NDV, IBV, ILTV, ARV, and FAdV4) were examined using the GeXP multiplex RT-PCR assay.

### Evaluation of the Sensitivity of the GeXP Multiplex RT-PCR Assay

The sensitivity of the GeXP assay was evaluated using *in vitro* transcribed RNAs for the N1 to N9 and M genes. Briefly, the nine NA genes and one M gene were amplified using the primers listed in Table 1, PCR amplicons were ligated into the pGEM-T vector, and expanded in competent DH5α cells to produce ten recombinant plasmids. The ten plasmids were purified, sequenced, linearized, and subjected to *in vitro* transcription according to the instructions of T7 RiboMAXTM Express Large Scale RNA Production System kit (Promega, Madison, WI, United States). The *in vitro* transcribed RNAs were quantified using a NanoDrop 2000 (Thermo Fisher Scientific); then, serial 10-fold dilutions were prepared. Ten premixed RNA templates at the same concentrations were prepared.

### Detection in Clinical Samples

Three hundred fifty swab samples (the oral pharyngeal and cloacal swabs from the same bird were pooled as a single sample) were obtained as part of AIV surveillance programs in live bird markets (LBMs) in Nanning, the capital of Guangxi Province, from 2016–2017. RNA was extracted from the washing solution of the swabs and analyzed according to the protocol established for the GeXP multiplex RT-PCR assay. All samples were inoculated in parallel in the 9-day-old SPF embryonated chicken eggs for virus isolation. Isolated allantoic fluids were identified using the neuraminidase (NA) assay and neuraminidase inhibition (NI) test, according to the World Health Organization (WHO) protocol^2^. The allantoic fluids were also amplified with conventional RT-PCR followed by NA amplicon sequencing.

## Results

### Evaluation of the Single Primers With the GeXP Mono-RT-PCR Assay

RNA samples extracted from nine NA subtypes of AIV were used as individual templates for GeXP mono-RT-PCR in separate reactions to evaluate the specificity of each pair of gene-specific primers. In the mono-RT-PCR assays, the pan-AIV primers amplified all AIV subtypes, and each pair of subtype-specific primers only generated a product for the NA gene corresponding to the target subtype.

### Screening of the Optimal Multiplex Primers

Amplicons were designed to ensure that each fragment was no less than 5 nucleotides away from its nearest neighbor and allowed for variation in peak migration to meet the minimum peak separation distance of 3 nucleotides. In this study, fragments of the expected sizes were amplified for the nine NA subtypes: N1, 244 to 249 bp; N2, 279 to 285 bp; N3, 215 to 221 bp; N4, 149 to 155 bp; N5, 295 to 301 bp; N6, 236 to 241 bp; N7, 192 to 198 bp; N8, 173 to 178 bp; N9, 205 to 211 bp; and AIV-M, 158 to 164 bp.

### Evaluation of the Multiplex Primers Using the GeXP Multiplex RT-PCR Assay With Templates for Single and Multiple Subtypes

**FIGURE 1:**
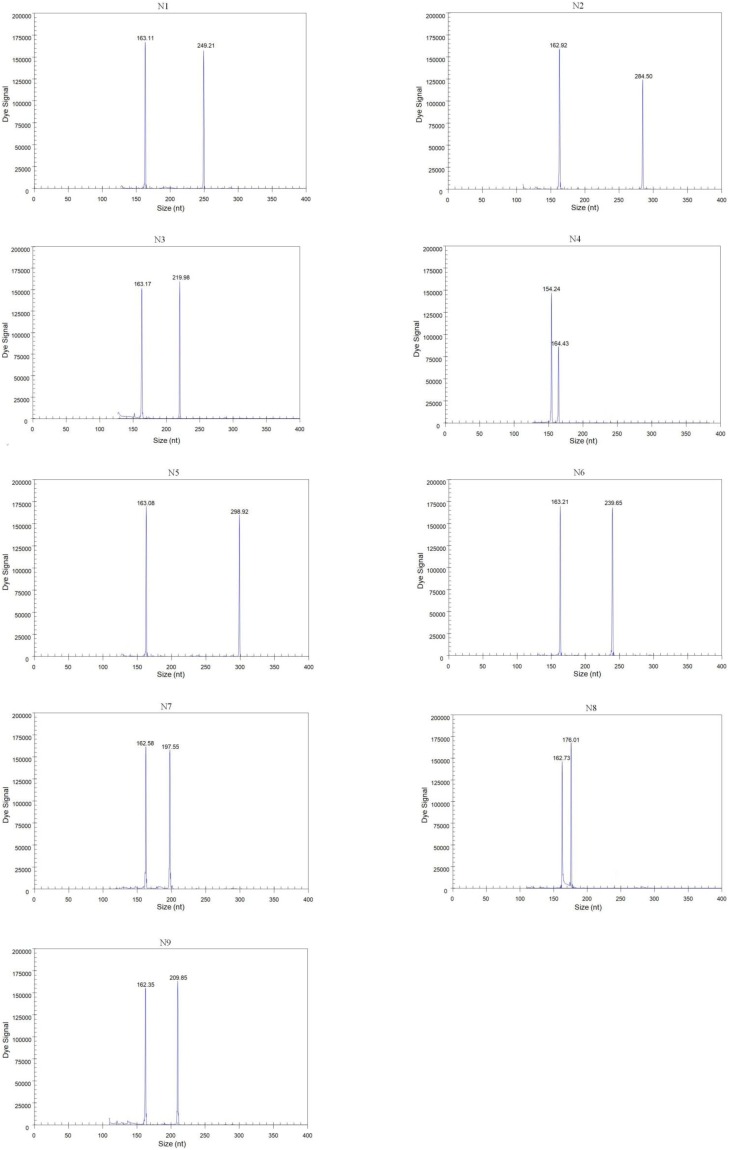
Detection of single AIV subtypes using GeXP multiplex RT-PCR. The *Y*-axis indicates the fluorescence signal in arbitrary units, and the *X*-axis indicates the actual size of the PCR product.

Forty-three reference isolates (Table 2), including N1 to N9 subtypes of the influenza A virus originating from different host species, such as duck, chicken, and pigeon, were subjected to GeXP multiplex RT-PCR. Two specific amplification peaks were observed, representing the subtype-specific target amplicon and the AIV M gene amplicon (Figure 1). No cross-reactivity among these nine subtypes was observed. Genomic RNA and DNA extracted from other important avian viruses, such as NDV, IBV, ILTV, ARV, and FAdV4, tested negative in the GeXP multiplex RT-PCR (data not shown). The high specificity of the GeXP multiplex RT-PCR assay described here was also confirmed by the investigation of well-characterized AIV reference viruses and non-AIV avian viruses. Each sample was assayed three times under the same conditions on different days and produced very similar results.

We randomly mixed RNAs from two different subtypes at different concentrations (10^2^ copies of one template and 10^6^ copies of the other template) as templates to assess the performance characteristics of GeXP for the diagnosis of a mixed infection with multiple NA subtypes. Regardless of whether a high or low concentration was used, the two subtype-specific peaks were both observed, with values similar to each template alone. Furthermore, templates with random mixtures of three or four subtypes (from 10^2^ to 10^6^ copies) were evaluated using the GeXP assay, and all target subtypes were accurately amplified. The amplification results for the multiplex templates were similar to the single-template amplifications, suggesting that this GeXP assay is useful for identifying different NA subtypes in a mixed sample. Each group was assayed three times under the same conditions on different days and produced very similar results.

### Sensitivity of the GeXP Multiplex RT-PCR Assay

**FIGURE 2:**
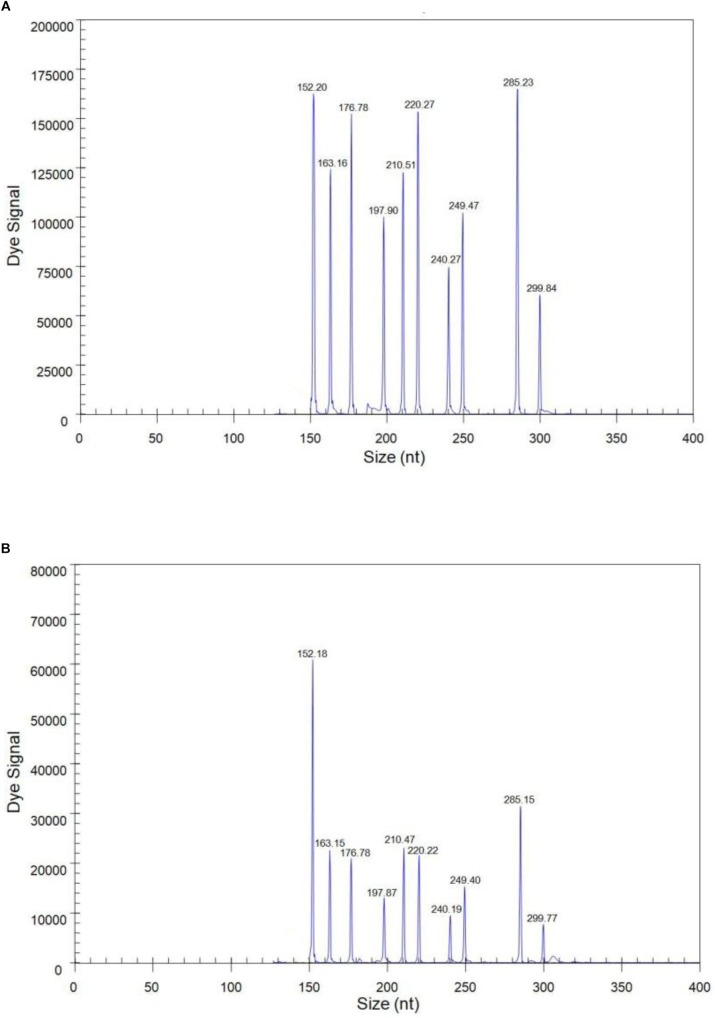
The sensitivity of the GeXP assay for the simultaneous detection of nine NA subtypes of AIV. All 10 premixed viral targets were detected. **(A)** 10^3^ copies per reaction mixture; **(B)** 100 copies per reaction mixture.

The sensitivity of the GeXP assay was measured using serial 10-fold dilutions of ten premixed quantitative *in vitro* transcribed RNAs from the viral targets. The limit of simultaneous detection of the ten templates of N1, N2, N3, N4, N5, N6, N7, N8, N9, and AIV using the GeXP multiplex RT-PCR assay was as low as 100 copies per reaction mixture (Figure 2). Typically, the cut-off CT value for positive and negative results was determined as 2000 A.U. (absorbance units) by default. Each sample was assayed three times under the same conditions on different days and produced very similar results.

### Detection in Clinical Samples

Among three hundred fifty samples, twenty-seven samples were positive and the remaining were negative for AIV and NA subtypes using three methods: GeXP, NA amplicon sequencing, and the NI test. Among the twenty-seven positive cases, two were identified as two subtypes of mixed infection using GeXP and the NI test, but amplicon sequencing only identified one subtype. Consistent results for the remaining twenty-five cases were obtained with these three methods. The results of NA subtyping are shown in Table 3.

**Table 3:** Results obtained from clinical samples using GeXP multiplex RT-PCR, the NI test, and amplicon sequencing method.

Number	Host	GeXP	NI assay	Amplicon sequencing
1	Chicken	N2	N2	N2
2	Chicken	N2	N2	N2
3	Chicken	N2	N2	N2
4	Chicken	N2	N2	N2
5	Chicken	N2	N2	N2
6	Chicken	N6	N6	N6
7	Chicken	N6	N6	N6
8	Chicken	N6	N6	N6
9	Chicken	N9	N9	N9
10	Duck	N1	N1	N1
11	Duck	N1	N1	N1
12	Duck	N2	N2	N2
13	Duck	N2	N2	N2
14	Duck	N2	N2	N2
15	Duck	N2/N6	N2/N6	N2
16	Duck	N6/N2	N6/N2	N6
17	Duck	N6	N6	N6
18	Duck	N8	N8	N8
19	Duck	N8	N8	N8
20	Duck	N8	N8	N8
21	Duck	N9	N9	N9
22	Duck	N2	N2	N2
23	Duck	N2	N2	N2
24	Duck	N2	N2	N2
25	Duck	N6	N6	N6
26	Duck	N6	N6	N6
27	Duck	N6	N6	N6

## Discussion

Recently, the reassortment of HA subtypes with multiple NA subtypes has increased; for example, H5N1, H5N2, and H5N3 were previously detected, and H5N6 and H5N8 are now emerging. H7 assorts with different NA subtypes (N2, N3, N4, N7, and N9 have been found in cases of human infection). H6N1, H6N2, H6N5, H6N6, and H6N8 were identified during the surveillance of live poultry markets in China (Peng et al., 2013). H4 and H3 reassort with N2, N6, and N8. H9 usually assorts with N2 (Luo et al., 2017). Epidemiological surveys of some viruses use only real-time PCR to analyze DNA/RNA from swab samples, with no further isolation. However, AIV is a segmented virus, and rearrangement of its eight gene segments during coinfection with two or more subtypes produces new recombinant strains. For an early warning of emerging flu outbreaks, we must understand the ecological evolution of AIV isolates. Therefore, surveys of AIV usually require the isolation of the virus followed by the selection of representative strains for gene sequencing and a subsequent genetic evolution analysis. Swab-inoculated SPF chick embryos are used for virus propagation, allantoic fluid is harvested and screened with an HA assay, and HA subtypes are identified with an HI test. Here, the confirmation of the NA subtype is important and a key step in the process. In addition, the routine monitoring of AIV frequently involves a large number of samples. Therefore, the development of this high-throughput GeXP assay for the simultaneous and rapid detection of these nine NA subtypes is important for the effective prevention and control of AIV.

In clinical samples, sequencing identified only a single NA subtype. This result was expected, because sequencing is performed on samples with positive single colony in the panel and is preceded by conventional RT-PCR to amplify a high concentration of NA subtypes. Thus, in the panel of clones and transformants, most single colonies should possess the dominant NA genes. Therefore, the determination of the existence of a mixed infection with different NA subtypes is difficult using amplicon sequencing, which guarantees only the detection of the dominant NA subtype. Certainly, amplicon sequencing occasionally, but not always, identifies different NA subtypes in different colonies. This uncertainty is a limitation of sequence identification based on amplicon cloning. However, GeXP, which simultaneously detects nine NA subtypes in a single tube and rapidly identifies any number of coinfecting NA subtypes, addresses this limitation. The ability of the GeXP assay to identify mixed infections was superior to clone sequencing.

In the traditional multiplex RT-PCR assay, multiplex primers compete for amplification, and the primers with a high amplification efficiency or smaller fragments are more likely to dominate and be preferred. As the number of primers increased, the probability of forming complex primer dimers also increases, leading to a decrease in sensitivity. In addition, the results of multiple PCR products must be observed using agarose electrophoresis, and bands less than 100 bp are difficult to distinguish. In the real-time multiplex RT-PCR assay, multiple probes are labeled with fluorescent dyes that are excited at different wavelengths. A greater number of fluorescent tags will increase the mutual interference and decrease the sensitivity. The number of target genes that can be detected using multiplex PCR and multiplex real-time PCR is limited, as only 2 to 4 pathogens are generally able to be detected, and neither assay achieves the purpose of rapid and high-throughput detection and analysis of multiple pathogens. The GeXP technology has recently overcome these shortcomings. In the present study, nine pairs of NA subtype-specific primers and one pair of pan-AIV primers were designed to develop a GeXP assay for the simultaneous identification of nine NA subtypes of AIV, including N1, N2, N3, N4, N5, N6, N7, N8, and N9. The pan-AIV primer was added to the set of primers to provide evidence that the samples were indeed AIV. The subtype-specific primers were designed to target the NA gene of each NA subtype, and the pan-AIV primer was designed to target the M gene, which is relatively conserved among subtypes. In the GeXP assay, which was based on both subtype-specific and universal primers, the forward and reverse universal primers were constant, and the universal primer was attached to the 5′ end of each specific primer. Another pair of universal primers was mixed in 5× GeXP PCR buffer, and the 5′ end of this universal forward primer was labeled with a fluorescent dye. At the beginning of the multiplex PCR process, specific primer sequences were combined with different NA subtype templates, and amplicons with universal sequence tags at both the 5′ and 3′ ends were generated. The amplification was quickly controlled by the universal primers, since the universal primers were present in the PCRs at significantly higher concentrations than the gene-specific primers. Amplification by multiplex primers was converted into amplification by a pair of universal primers, and thus the amplification efficiency of each template tended to be concordant, without effects on the amplification efficiency of each pair of primers. This method results in the highly sensitive and specific amplification of different genes in a single multiplex RT-PCR assay, preventing preferred and inferior amplification, and minimizing non-specific reactions. PCR products were separated using capillary gel electrophoresis, and the separated fragments were initially analyzed using the fragment analysis module of the GeXP system software. The amplified fragments were visualized as fluorescence signal peaks and different gene fragments were identified by size. Despite the limited number of positive samples for the other four subtypes of AIV (N3, N4, N5, and N7), we propose that the methods described here could be extended to the routine diagnosis and epidemiological detection of AIV infections.

The GeXP assay developed here simultaneously differentiated nine NA subtypes in a single reaction tube with a cost of approximately $8 per comprehensive test compared with $8 per test for each subtype using a commercial real-time RT-PCR kit. The whole reaction, including RNA extraction, was completed in one tube in a multiplex PCR system within 3 h, followed by a capillary electrophoresis separation step lasting approximately 1 h. In addition, two 96-well plates can be simultaneously placed in parallel in a GeXP machine to further increase sample throughput. Moreover, the procedure is easily automated and requires minimal hands-on time by technical staff. In conclusion, this GeXP assay is proposed as a specific, sensitive, rapid, high-throughput, and versatile diagnostic tool for nine NA subtypes of AIV. In the future, we will develop additional methods to simultaneously detect important HA and NA subtypes of AIV.

## Data Availability

The raw data supporting the conclusions of this manuscript will be made available by the authors, without undue reservation, to any qualified researcher.

## Author Contributions

ZXX designed the experiments and supervised the study and helped to review the manuscript. SL, JH, ZQX, LX, MZ, ML, SW, and DL performed the experiments. SL, ZXX, TZ, YZ, QF, and XD analyzed the data. SL wrote the manuscript.

## Conflict of Interest Statement

The authors declare that the research was conducted in the absence of any commercial or financial relationships that could be construed as a potential conflict of interest.

## Footnotes

1 https://www.ncbi.nlm.nih.gov/genomes/FLU/Database/nph-select.cgi

2 https://docplayer.net/15666727-Who-global-influenza-surveillance-network-manual-for-the-laboratory-diagnosis-and-virological-surveillance-of-influenza.html
